# Germline mutations in the spindle assembly checkpoint genes *BUB1* and *BUB3* are infrequent in familial colorectal cancer and polyposis

**DOI:** 10.1186/s12943-018-0762-8

**Published:** 2018-02-15

**Authors:** Pilar Mur, Richarda M. De Voer, Rubén Olivera-Salguero, Sandra Rodríguez-Perales, Tirso Pons, Fernando Setién, Gemma Aiza, Rafael Valdés-Mas, Angelo Bertini, Marta Pineda, Lilian Vreede, Matilde Navarro, Silvia Iglesias, Sara González, Joan Brunet, Alfonso Valencia, Manel Esteller, Conxi Lázaro, Geert J. P. L. Kops, Miguel Urioste, Xose S. Puente, Gabriel Capellá, Laura Valle

**Affiliations:** 1 Hereditary Cancer Program, Catalan Institute of Oncology, IDIBELL, Hospitalet de Llobregat, Av. Gran Via 199-203, 08908 Barcelona, Spain; 2 Program in Molecular Mechanisms and Experimental Therapy in Oncology (Oncobell), IDIBELL, Hospitalet de Llobregat, Barcelona, Spain; 3 Centro de Investigación Biomédica en Red de Cáncer (CIBERONC), Salamanca, Spain; 4 Department of Human Genetics, Radboud Institute for Molecular Life Sciences, Radboud University Medical Center, Nijmegen, The Netherlands; 5 Hubrecht Institute – KNAW (Royal Netherlands Academy of Arts and Sciences), Utrecht, The Netherlands; 6 Molecular Cytogenetics Group, Human Cancer Genetics Program, Spanish National Cancer Research Center (CNIO), Madrid, Spain; 7 Structural Biology and Biocomputing Program, Spanish National Cancer Research Center (CNIO), Madrid, Spain; 8 Department of Immunology and Oncology, National Centre for Biotechnology (CNB-CSIC), Madrid, Spain; 9 Cancer Epigenetics and Biology Program (PEBC), Bellvitge Biomedical Research Institute (IDIBELL), Hospitalet de Llobregat, Barcelona, Spain; 10 Department of Biochemistry and Molecular Biology, Instituto Universitario de Oncología del Principado de Asturias, Universidad de Oviedo and CIBERONC, Oviedo, Spain; 11 Hereditary Cancer Program, Catalan Institute of Oncology, IDIBGi, Girona, Spain; 12 Life Science Department, Barcelona Supercomputing Centre (BSC-CNS), Barcelona, Spain; 13 Institució Catalana de Recerca i Estudis Avançats (ICREA), Barcelona, Spain; 14 Physiological Sciences Department, School of Medicine and Health Sciences, University of Barcelona (UB), Barcelona, Spain; 15 Familial Cancer Clinical Unit, Human Cancer Genetics Program, Spanish National Cancer Research Centre (CNIO) and Center for Biomedical Network Research on Rare Diseases (CIBERER), Madrid, Spain

**Keywords:** Colorectal cancer predisposition, Hereditary colorectal cancer, High-penetrance genes, Variegated aneuploidy, Mechanism

## Abstract

Germline mutations in *BUB1* and *BUB3* have been reported to increase the risk of developing colorectal cancer (CRC) at young age, in presence of variegated aneuploidy and reminiscent dysmorphic traits of mosaic variegated aneuploidy syndrome. We performed a mutational analysis of *BUB1* and *BUB3* in 456 uncharacterized mismatch repair-proficient hereditary non-polyposis CRC families and 88 polyposis cases. Four novel or rare germline variants, one splice-site and three missense, were identified in four families. Neither variegated aneuploidy nor dysmorphic traits were observed in carriers. Evident functional effects in the heterozygous form were observed for c.1965-1G>A, but not for c.2296G>A (p.E766K), in spite of the positive co-segregation in the family. *BUB1* c.2473C>T (p.P825S) and *BUB3* c.77C>T (p.T26I) remained as variants of uncertain significance. As of today, the rarity of functionally relevant mutations identified in familial and/or early onset series does not support the inclusion of *BUB1* and *BUB3* testing in routine genetic diagnostics of familial CRC.

**Electronic supplementary material:**

The online version of this article (10.1186/s12943-018-0762-8) contains supplementary material, which is available to authorized users.

## Letter to the editor

Much of the heritability associated with colorectal cancer (CRC) cannot be explained by the currently known genetic risk factors for CRC. Genome-wide genetic and genomic screenings have tried to identify novel high penetrance genes for CRC. Despite the identification of novel candidate genes for CRC predisposition, overall, they account for a very low number of familial cases, and for many of the suggested genes, identification of additional mutated families is essential to conclusively define their actual implication in CRC predisposition [1].

Based on genome-wide copy number profiling and exome sequencing in early-onset and familial CRC, De Voer et al. identified germline mutations in *BUB1* and *BUB3*, components of the spindle assembly checkpoint (SAC) and thus controllers of correct chromosome segregation, as cause for CRC predisposition [2, 3]. Six germline mutations affecting *BUB1* and *BUB3* were identified in 6 independent families. Recently, Broderick et al. assessed the presence of germline mutations in proposed genes for CRC predisposition, including *BUB1* and *BUB3*, finding no increased frequency of mutations in cases compared to controls in either gene [4]. In view of the controversial results, the purpose of the present study is to evaluate the impact, supported by functional studies, of *BUB1* and *BUB3* germline variants in the predisposition to CRC and/or polyposis.

**Fig. 1:**
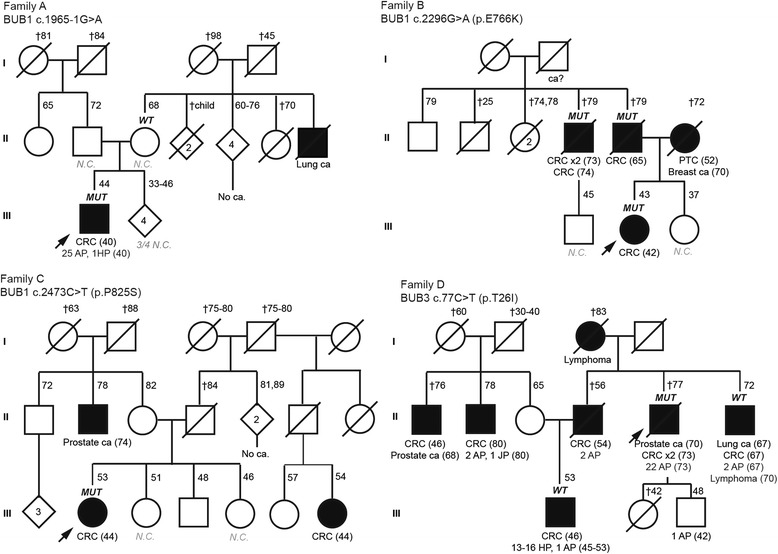
Pedigrees of the families with *BUB1* and *BUB3* mutations. Filled symbol, cancer. Ages at information gathering or at death (†), when available, are indicated on the top-right corner, and ages at cancer diagnosis, between brackets after tumor type. Abbreviations: AP, adenomatous polyp; HP, hyperplastic polyp; JP, juvenile polyp; ca, cancer; CRC, colorectal cancer; PTC, papillary thyroid cancer; N.C., normal colonoscopy; *MUT*, mutation carrier; *WT* (wildtype); non-carrier of the mutation identified in the family

Using a strategy that combines pooled DNA amplification and massively parallel sequencing, we sequenced *BUB1* and *BUB3* in 456 familial colorectal cancer cases (60 Amsterdam-positive families), and in 88 polyposis cases, without identified mutations in known high-penetrance genes. Considering novel and rare (population minor allele frequency (MAF) < 1%) non-synonymous genetic changes, a total of 4 variants, *BUB1* c.1965-1G>A, *BUB1* c.2296G>A (p.E766K), *BUB1* c.2473C>T (p.P825S) and *BUB3* c.77C>T (p.T26I), were detected in 4 families (Table 1, Fig. 1). Only *BUB1* c.2473C>T was reported in public databases. Loss of heterozygosity (LOH) causing the elimination of the wild-type allele or promoter methylation was not detected in any of the tumors studied (Table 1, Additional file 1: Table S1), as previously observed [2].

**Table 1:** Germline genetic variants in *BUB1* and *BUB3* identified in 544 CRC and/or polyposis families

^a^Mutation [protein domain]	BUB1 c.1965-1G>A (r.1965_1975del; p.S655Rfs*32)	BUB1 c.2296G>A (p.E766K)	BUB1 c.2473C>T (p.P825S) [Kinase domain]	BUB3 c.77C>T (p.T26I) [WD40 repeat 1]
Population MAF (%) (1000G / ESP / ExAC/ gnomAD)	n.r.	n.r.	rs748392521; n.r. / n.r. / 0.004 / 0.005	n.r.
Protein function prediction (score)	p.S655Rfs*32	^b^PPH2: PsD (0.934) / N (0.349)	^b^PPH2: PrD (1 / 0.997)	^b^PPH2: PsD (0.770 / 0.548)
		SIFT: N (0.2)	SIFT: D (< 0.0001)	SIFT: N (0.06)
		Condel: N (0.463)	Condel: D (0.542)	Condel: D (0.524)
		Mut. Taster: D	Mut. Taster: D	Mut. Taster: D
		Align GVGD: N (C0)	Align GVGD: D (C65)	Align GVGD: N (C0)
Protein stability prediction (ddG, Kcal/mol)	p.S655Rfs*32	CUPSAT: destabilizing (−0.69)	CUPSAT: destabilizing (−1.16)	CUPSAT: destabilizing (− 0.29)
		ERIS: destabilizing (1.57)	ERIS: stabilizing (−0.71)	ERIS: destabilizing (1.10)
		I-Mutant: destabilizing (−0.53)	I-Mutant: destabilizing (−0.64)	I-Mutant: destabilizing (− 0.68)
		PoPMuSIC: destabilizing (0.04)	PoPMuSIC: stabilizing (0.17)	PoPMuSIC: destabilizing (0.33)
Splice site predictions (Alamut v2.9)	Disrupts acceptor site	No change	No change	No change
	RNA study: 11 bp deletion	RNA study: no change		RNA study: no change
^c^Evolutionary conservation (PhyloP / PhastCons)	–	1.239 / 0.95	4.358 / 1	5.572 / 1
Variegated aneuploidy	No	No	n.a.	No
Somatic LOH / promoter methylation	n.a. / n.a.	No LOH; No methylation (II.6 and III.2)	No LOH; No methylation	No LOH; n.a.
Functional studies in LCL	Damaging effects	No effect (incomplete functional assessment)	n.a.	n.a.
^d,e^Variant classification	^e^Pathogenic	Likely benign / Uncertain significance	Uncertain significance	Uncertain significance

a. RefSeq GRCh37: BUB1 NM_004336, BUB3 NM_004725

b. Polyphen 2: HumDiv / HumVar scores.

c. PhyloP score (values between -14 and +6): Sites predicted to be conserved are assigned positive scores. PhastCons score (values between 0 and 1): the closer the value is to 1, the more probable the nucleotide is conserved.

d. According to the standard guidelines for the interpretation of sequence variants (recommendations of the American College of Medical Genetics and Genomics and the Association for Molecular Pathology) [7].

e. Interpret with caution. BUB1 and BUB3 have not yet been defined as genes with clinical value in hereditary cancer.

Abbreviations: 1000G, 1000 Genomes; bp, base pairs; ca., cancer; D, damaging or deleterious; ESP, NHLBI GO Exome Sequencing Project; ExAC, Exome Aggregation Consortium; GnomAD, Genome Aggregation Database (http://gnomad.broadinstitute.org); LCL, EBV-transformed lymphoblastoid cell line; N, neutral; n.a., not available; n.r., not reported; PPH2, polyphen 2; PrD, probably damaging; PsD, possibly damaging

**Fig. 2:**
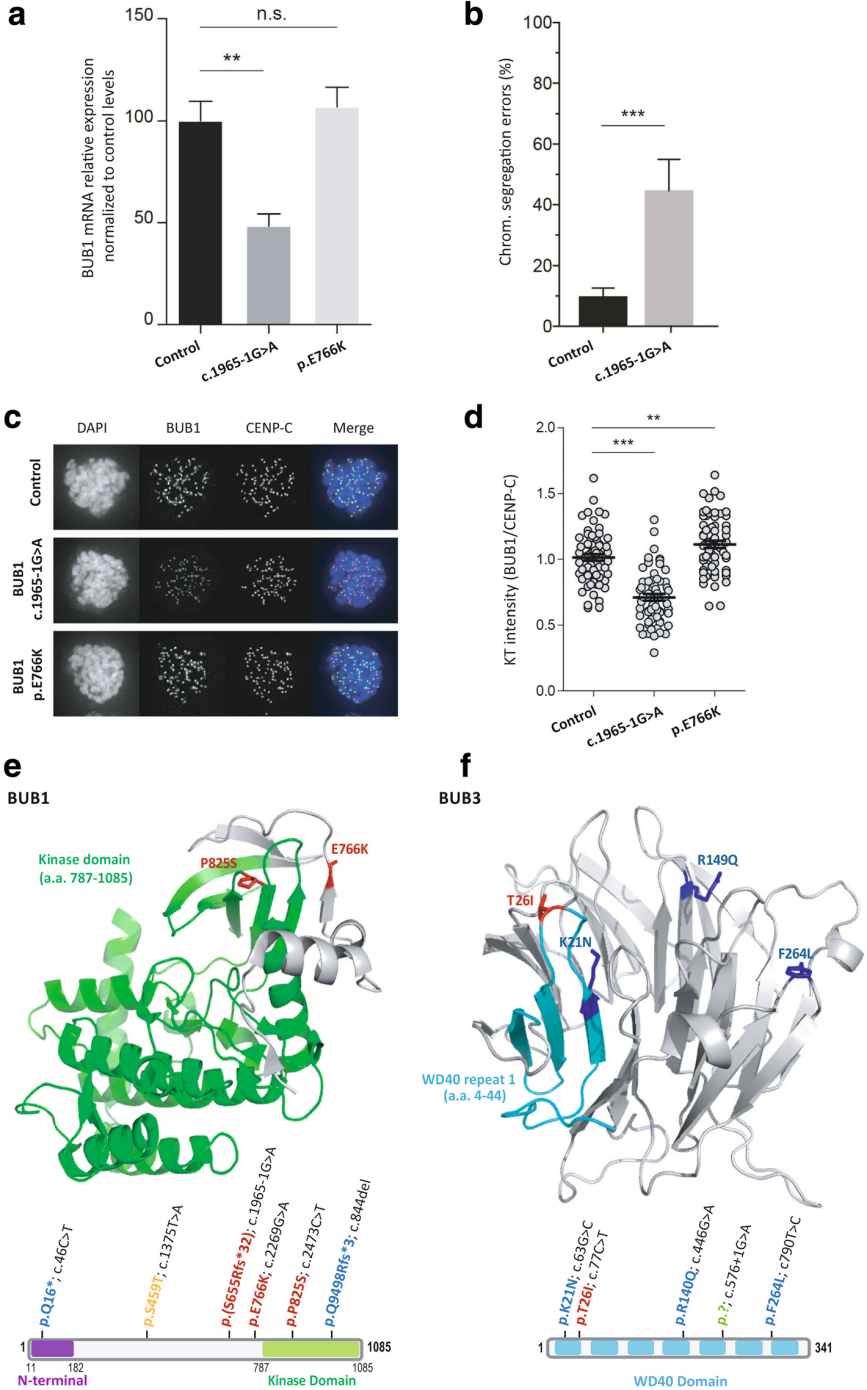
Functional assays for *BUB1* c.1965-1G>A and c.2296G>A (p.E766K) and 3D structure of the proteins and location of the identified *BUB1* and *BUB3* germline mutations. **a** Quantitative analysis of levels of *BUB1* mRNA demonstrating reduced *BUB1* expression in the c.1965-1G>A cell line compared to controls. Data are normalized against *HPRT* expression. Error bars represent standard error mean (SEM) values. **b** Quantification of chromosome segregation errors in EBV-transformed cells from a control individual and the *BUB1* c.1965-1G>A mutation carrier. Measurements were performed in triplicate and error bars represent SEM values. **c** Localization and **d** quantification of BUB1 levels at the kinetochores of nocodazole-arrested EBV-transformed cells. Each dot represents one cell and the level of BUB1 is normalized to the kinetochore (KT) intensity of CENP-C and is the average fold change of three experiments (±SEM) normalized to the values of control cells. Cells from the c.1965-1G>A carrier revealed reduced levels of BUB1 at the KT in comparison to the control. ** *P* < 0.001; *** *P* < 0.0001. **e** Crystallographic 3D structure of BUB1 (a.a. 736–1083) and location of p.E766K and p.P825S, and 3D model of BUB3 (a.a. 6–324) and location of p.T26I (current study) and p.K21N, p.R149Q and p.F264L [2]. **f** Protein domains of BUB1 (UniProtKB - O43683) and BUB3 (UniProtKB - O43684) and location of the identified mutations. In red, residues affected by mutations identified in this study; in *blue*, residues affected by the mutations identified by de Voer et al. [2]; in orange, variants identified by Broderick et al. [4]; and in green, variants detected by Shindo et al. [6]

*BUB1* c.1965-1G>A, identified in a male patient diagnosed with CRC and 25 adenomatous polyps at age 40 with no relevant family history of cancer (Fig. 1), causes an out-of-frame deletion of 11 bases, produced by the disruption of the canonical acceptor site of exon 18 and usage of the next AG as novel splicing acceptor (r.1965_1975del; p.S655Rfs*32) (Additional file 1: Figure S1). The EBV-transformed lymphoblastoid cell line obtained from this carrier showed reduced *BUB1* mRNA expression (*p* = 0.0020) and increased chromosome segregation errors (*p* = 0.0050) compared to control lymphoblastoid cell lines (Fig. 2a-b). The c.1965-1G>A cells showed reduced BUB1 levels at the kinetochores, where the SAC is active, compared to controls (30% reduction; *p* < 0.0001) (Fig. 2c-d).

*BUB1* c.2296G>A (p.E766K), located in a β-strand outside the protein kinase domain, was identified in three CRC-affected members of an Amsterdam-positive CRC family (Fig. 1). Computational analyses predicted a destabilizing effect on the protein (Table 1). However, the EBV-transformed lymphoblastoid cell line obtained from the proband did not show reduced BUB1 expression (Fig. 2a) or reduced BUB1 levels at the kinetochores (Fig. 2c-d) when compared to controls. Assessment of chromosome segregation errors could not be performed.

*BUB1* c.2473C>T (p.P825S) (rs748392521, MAF_ExAC_: 0.004%), which affects a highly conserved amino acid (considering 13 species) and is located in the catalytic kinase domain of the protein, was identified in an individual diagnosed with CRC at age 44, with no immediate relatives affected with cancer but with a cousin diagnosed with CRC at age 44 whose mutation status could not be assessed (Fig. 1). The variant was predicted to affect the function and structure of the protein (Table 1, Fig. 2e and f). Sample unavailability prevented us from carrying out the specific functional studies in the patient-derived lymphoblastoid cell line.

Finally, *BUB3* c.77C>T (p.T26I) was identified in patient diagnosed with prostate cancer at age 70 and two synchronous CRCs and 22 adenomatous polyps at age 73, belonging to a family fulfilling the Amsterdam I criteria. The variant, located in the WD40 repeat 1 of a seven-bladed beta-propeller fold, was predicted to be functionally deleterious and destabilizing (Table 1). The location of the mutated residue together with the previously reported *BUB3* CRC-predisposing mutations is shown in Fig. 2e-f. Co-segregation results in other cancer-affected family members did not support a causal role of the variant in the aggregation of cancer in the family (Fig. 1). The in vitro functional studies could not be performed due to the unsuccessful transformation of the patient’s lymphocytes.

Cytogenetic analysis of non-transformed lymphocytes obtained from *BUB1* c.1965-1G>A, *BUB1* c.2296G>A (p.E766K), *BUB3* c.77C>T (p.T26I) mutation carriers and from unrelated controls, did not show differences in the number of metaphases with chromosome number alterations. Notably, complex chromosomal translocations were identified in one of 41 metaphases of the *BUB1* c.1965-1G>A carrier, while no chromosomal translocations were detected in any of the 91 control metaphases analyzed (Additional file 1: Table S2).

No reminiscent traits of the mosaic variegated aneuploidy syndrome or variegated aneuploidy in lymphocytes were found in any of the studied carriers, not even in the carrier of *BUB1* c.1965-1G>A (p.S655Rfs*32). However, the EBV-transformed lymphocytes from the latter revealed chromosome segregation errors, which may lead to aneuploidy in the next cell cycle. It is unclear what the cell fate of the missegregated cells is, as the subsequent chromosomal instability might be detrimental. De Voer et al. identified mosaic aneuploidy in all three *BUB1*/*BUB3* mutation carriers they studied, while only two of the three carriers showed dysmorphic features [2]. On the other hand, no aneuploidies were observed in a 54 year-old Dupuytren patient without family history of CRC and a 1.7 Mb deletion of chromosome 2q13, which includes *BUB1* [5]. These findings suggest that *BUB1/3* monoallelic mutations may or not cause mosaic aneuploidy and/or phenotypic affectation.

The role of *BUB1* and *BUB3* in familiar cancer has been topic of debate in the last years. Recently, Broderick et al. scrutinized the exomes of 863 familial/early-onset CRC cases and 1604 cancer-free controls in order to validate the proposed hereditary CRC genes, including *BUB1* and *BUB3* [4]. Neither the herein identified variants nor the ones identified by De Voer et al. [2] were detected in the exome study. While only one novel/rare missense variant predicted to be deleterious (none stop-gain, frameshift or splice-site variants) was identified in *BUB1* in the 863 cases (0.11%), and none in *BUB3*, several (4 in *BUB1* (0.25%) and 2 in *BUB3* (0.12%)) novel/rare disruptive variants were identified in controls. Of note, the frequency of disruptive (stop-gain, frameshift and splice acceptor/donor) mutations in controls identified by Broderick et al. is remarkably higher than the frequency of disruptive mutations annotated in large population-based browsers (*BUB1*: ExAC, 0.065% (40 carriers in 60,703 individuals); GnomAD, 0.063% (88/138,044). *BUB3*: ExAC, 0.006% (4/59,569); GnomAD, 0.008% (11/123,084)) [4]. Taking into account the two largest series analyzed ([4] and present study), 0.14% (2/1392; 1/863 and 1/529, respectively) of familial and/or early-onset CRC cases would be explained by functionally relevant germline mutations in *BUB1* (0% in *BUB3*), compared to a population frequency of 0.063–0.065%. Nevertheless, despite the demonstrated functional effect of several *BUB1*/*BUB3* variants, their causal implication in colorectal carcinogenesis, either alone or in combination with other mutations/variants in other genes, is yet to be proven.

To date, 11 CRC families and 1 pancreatic ductal adenocarcinoma family with different novel/rare germline mutations in *BUB1* or *BUB3* have been reported (Additional file 1: Table S3; Fig. 2e-f) [2, 4, 6]. Of the 12 variants, 7 were proven deleterious based on functional experimental evidence and/or on their splice-site or frameshift nature, one did not show any effect, and the remaining 4 missense variants were not subjected to functional studies. Six of the 8 mutation carriers (7 families) in whom functionally relevant mutations have been identified were diagnosed with CRC at young ages (range: 29–40 years), one of them together with the presence of 25 adenomas. The increased risk to other tumor types, such as lung cancer, remains uncertain until additional functionally relevant mutation carriers with the disease are identified.

Based on the evidence gathered to date, we conclude that the paucity of functionally-relevant cancer-predisposing mutations in *BUB1* and *BUB3* do not support the need for germline genetic testing of these genes in familial CRC for diagnostic purposes.

## Additional file

Additional file 1: Methods and Supplementary information (DOC 291 kb)

## Abbreviations

a.a.: amino acid
CRC: colorectal cancer
EBV: Epstein Barr virus
ExAC: Exome Aggregation Consortium (http://exac.broadinstitute.org/)
GnomAD: Genome Aggregation Database (http://gnomad.broadinstitute.org/)
LOH: loss of heterozygosity
MAF: minor allele frequency
SAC: spindle assembly checkpoint
SEM: standard error mean
