# Supplementary material for: Germline mutations in the spindle assembly checkpoint genes BUB1 and BUB3 are infrequent in familial colorectal cancer and polyposis
Source: Mol Cancer. 2018 Feb 15;17:23. doi: 10.1186/s12943-018-0762-8 (PMC5815240; doi:10.1186/s12943-018-0762-8)
Supplement: Supplementary file 1 — Methods and Supplementary information (DOC 291 kb) [file 12943_2018_762_MOESM1_ESM.doc]

**METHODS AND SUPPLEMENTARY INFORMATION**

**METHODS**

**Patients**

A total of 544 CRC cases belonging to 529 families without mutations in previously reported high-penetrance CRC genes, were included in the study: 456 familial CRC cases from 441 uncharacterized MMR-proficient families, including 60 Amsterdam-positive families, and 88 unrelated polyposis cases [1]. Patients were assessed at the Genetic Cancer Counseling Units of the Catalan Institute of Oncology and the Spanish National Cancer Research Center (CNIO), both in Spain, between 1999 and 2012. Informed consent was obtained from all subjects and the study received the approval of the Ethics Committees of the Bellvitge Biomedical Research Institute (IDIBELL) (PR073/12).

**DNA and RNA extraction**

Genomic DNA from peripheral blood was extracted by using the FlexiGene DNA kit (Qiagen) and from formalin-fixed paraffin-embedded samples, with the QIAamp DNA FFPE tissue Kit (Qiagen, Hilden, Germany), both following the manufacturer’s instructions. Total RNA from lymphocytes was extracted using a standard Trizol-based protocol, and cDNA was synthesized using the Transcriptor First strand cDNA synthesis Kit (Roche Diagnostics GmbH, Mannheim, Germany).

**Mutation identification in pooled samples**

Patients were screened for *BUB1* and *BUB3* mutations using a combination of PCR amplification in pooled DNAs and targeted massively parallel sequencing, as previously described [2]. Amplification of the DNA pools was performed with Phusion High-Fidelity DNA Polymerase (New England Biolabs, Ipswich, MA, USA) and custom-designed primers (Table S4). Equimolar amounts of each amplicon were pooled, ligated and fragmented using a Covaris S2 (Covaris, Inc. MS, USA), and DNA libraries were prepared following the paired-end sample preparation protocol from Illumina (Illumina, Inc. CA, USA). Next generation sequencing was carried out on a HiSeq-2000 at the *Centro Nacional de Análisis Genómico* (CNAG, Barcelona, Spain). Variant identification was performed as previously described [2], and common variants present in dbSNP141 or 1000Genomes with a minor allele frequency higher that 1% were considered polymorphisms.

**Direct automated sequencing**

Sanger sequencing of the affected exon was performed to identify the mutated individuals among the samples included in the corresponding DNA pool, and for co-segregation studies in the families. Sequencing was performed on an ABI Sequencer 3730 using a standard protocol and data was analyzed with SeqMan Pro (Lasergene 13, DNASTAR) and Mutation Surveyor v.3.10 (SoftGenetics).

**Computational analyses**

Prediction of deleterious effect of the identified variants was performed by using: 1) splice-site analysis; 2) prediction of variant pathogenicity; 3) prediction of protein stability changes and 4) functional annotations that overlap or are in close proximity to the affected residues.

The potential effects on splicing were evaluated using the Alamut Visual v2.9 software (Interactive Biosoftware, Rouen, France), with includes the information derived from the following prediction programs/algorithms: NNSplice, MaxEnt, SSF, Human Splicing Finder (HSF) and GeneSplicer. The impact of missense variants at the protein level was analysed using SIFT, PolyPhen-2, CONDEL and Align-GVGD [3-7].

The crystallographic 3D structure of human BUB1 (a.a. 736-1083, PDB ID: 4R8Q chain A) was retrieved from the Protein Data Bank (http://www.rcsb.org/pdb/). A 3D-model of human BUB3 (a.a. 6-324) was obtained from ModBase (http://modbase.compbio.ucsf.edu/) and improved by the RepairPDB and Optimize commands of FoldX (http://foldx.crg.es). This model was calculated using as template the crystallographic structure of human mRNA export factor (Rae1) (PDB ID: 3mmy chain A), which shares 36% sequence identity with human BUB3 identity.

The effect on protein stability of the non-synonymous missense mutations identified was predicted with PoPMuSiC, Eris, I-Mutant 2.0 and CUPSAT [8-11]. Functional annotations (e.g. protein domains, known somatic mutations in different types of cancer, UniProt annotations from missense variants) that overlap the variant position, neighbor residues in close physical proximity, or affecting protein interaction interfaces, were retrieved with Structure-PPI (http://structureppi.bioinfo.cnio.es/Structure) [12].

**Culture of lymphocytes and splicing analysis**

Human lymphocytes from *BUB1* c.1965-1G>A, *BUB1* c.2296G>A and *BUB3* c.77C>T mutation carriers and two controls were cultured in the presence and absence of puromycin, as previously described [13]. PCR amplification of the synthesized cDNA was performed for the *BUB1* regions comprised between exons 17-19 for the study of c.1965-1G>A and between exons 19-20 for c.2296G>A. Exons 1-3 of *BUB3* were amplified for c.77C>T*.* PCR products were run in a 1.5% agarose gel and visualized in a UV transilluminator. Amplification products were sequenced. Primer sequences are detailed in Table S4.

**Aneuploidy study**

Heparinized blood samples were cultured for 72 hours in RPMI-1640 (Lonza) supplemented with fetal bovine serum (Invitrogen), gentamycin (Gibco) and phytohemaglutinin (Invitrogen) as mitotic activating factor. To visualize chromosomes, cells were exposed to colcemid (Gibco) to arrest cell division at metaphase. Cells were treated with a hypotonic solution for 20-30 min at 37ºC and then were fixed with freshly prepared Carnoy’s solution. For GTG banding, slides were trypsinized (Invitrogen) and stained by Giemsa (Invitrogen). Metaphase spreads were scored for aneuploidies using the Ikaros Metasystem software attached to a Zeiss Axioplan microscope. Healthy controls simultaneously processed with the samples were also studied. An average of 29 (range: 16-41) metaphases per case and 30 (range: 25-40) metaphases per control, were analyzed and GTG banding evaluated.

**Loss of heterozygosity**

Loss of heterozygosity (LOH) was studied on DNA extracted from paraffin-embedded tumor tissue and compared with DNA extracted from normal colon tissue or peripheral blood using microsatellite markers flanking the corresponding gene and covering a region of 2.8 Mb for *BUB1* (D2S1888, D2S1889, D2S1892) and of 2.6 Mb for *BUB3* (D10S1483, D10S587, D10S1723), or by Sanger sequencing. Primer sequences are detailed in Table S4.

**Promoter methylation analysis**

Genomic DNA extracted from formalin-fixed paraffin-embedded tissue was subjected to bisulfite treatment using the EZ DNA Methylation-Gold Kit (Zymo Research, Orange, CA, USA). The promoter region of *BUB1* (-212 to 35), containing 22 CpG sites, was amplified and subsequently sequenced using the bisulfite-converted DNA obtained from tumor and normal (blood or normal colon mucosa) tissue of the same patient, in order to identify tumor-specific promoter (CpG island) methylation. Primer sequences are shown in Table S4.

***BUB1* mRNA expression analysis in lymphoblastoid cell lines**

Lymphoblastoid cell lines (LCL) were generated by Epstein-bar virus transformation of lymphocytes obtained from *BUB1* c.1965-1G>A and *BUB1* c.2296G>A mutation carriers. LCLs were cultured in RPMI-1640 (Invitrogen) supplemented with 15% fetal calf serum, penicillin (100 U/ml), streptomycin (100 μg/ml) and HEPES (complete medium). Total mRNA was extracted using the RNeasy Mini Kit (Qiagen) according to manufacturer’s protocol. Total mRNA was reverse-transcribed using the RNA LA PCR kit (AMV, Takara Bio Inc, Shiga, Japan) with oligo(dT) and random 9-mers. Real-time quantification was performed on a 7500 Fast Real-Time PCR system (Applied Biosystems, CA, USA) using SYBR Green-based quantification (Promega). Experiments were performed in triplicate and data were normalized to *HPRT* expression. Relative expression was quantified using the 2^-ΔΔCt^ method.

**Mitotic checkpoint analysis**

Lymphoblastoid cell lines from *BUB1* c.1965-1G>A and *BUB1* c.2296G>A mutation carriers and controls were treated for 24 hours with 2 mM thymidine to synchronize cells in early S-phase and released from thymidine by four washes with complete medium. Cells were incubated with 50 nM SiR-DNA dye (Spirochrome) for 4.5 hours. Next, cells were seeded in 8-well Ibidi slides in 0.15% Ultrapure agarose, covered with 0.3% agarose and 50 nM SiR-DNA dye and 0.83 μM nocodazole in complete medium. Mitotic progression was assayed by acquiring images in a heated chamber (37°C and 5% CO_2_) every 5 minutes on a Nikon Ti-Eclipse widefield microscope equipped with an Andor Zyla 4.2 sCMOS camera, 40x oil objective NA 1.3 WD 0.2 mm, and Lumencor SpectraX light engine as described previously [14]. Images were acquired in 15 x 1.5 μm z layers and projected by maximum intensity projection into a single layer using NIS-Elements software 4.45. Single layer images were analyzed using ImageJ software and the time in mitosis was defined as the time between chromosome condensation and anaphase onset for each cell progressing through mitosis.

**Chromosome segregation analysis**

LCLs from *BUB1* mutation carriers and controls were transduced with a lentiviral construct encoding mNEON-green tagged histone 2B (H2B) and a puromycin-resistance cassette (pLV-H2B-mNeon-ires- Puro) as described previously [14, 15]. LCLs were synchronized for 24 hours in 2mM thymidine and released from S-phase as described above. After 4.5 hours LCLs were seeded in 8-well Ibidi slides in 0.15% Ultrapure agarose, covered with 0.3% agarose and complete medium. Chromosome segregation was assayed by acquiring images in a heated chamber (37°C and 5% CO_2_) every 3 minutes on a Nikon Ti-Eclipse microscope equipped with CSUW spinning disk (Yokogawa), Borealis, Andor iXon Ultra 888 EMCCD camera, 40x water objective NA 1.15 WD 0.6 mm, and 488 nm laser. Images were acquired in 15 x 1.5 μm z layers and projected by maximum intensity projection into a single layer using NIS-Elements software. Single layer images were analyzed with ImageJ. Cells progressing through mitosis were analyzed and the percentage of cells with chromosome segregation errors, such as multipolar mitoses, misaligned chromosomes, lagging chromosomes and chromosome bridges was determined.

**Immunofluorescence**

LCLs from BUB1 mutation carriers and controls were synchronized for 24 hours in 2 mM thymidine and released from S-phase as described and immediately seeded into complete medium with 3.3 μM nocodazole. After 5 hours, cells were seeded into complete medium with 3.3 μM nocodazole and 5 μM MG132 onto poly-L-lysine-coated coated 12 mm round coverslips. After 1 hour, cells were treated for 1 min with 0.1% Triton X- 100 in PEM buffer (100mM PIPES (pH 6.8), 5mM EGTA and 1mM MgCl_2_) and fixed with 50% zinc formalin fixative (Sigma) in PEM with 0.1% Triton X-100 for 10 minutes. Pre-extracted cells were washed with cold PBS twice and blocked with 3% BSA in PBS for 1 hour at RT. Subsequently, coverslips were incubated with primary antibodies in 3% BSA in PBS overnight at 4°C. Coverslips were washed 4x with 0.1% Triton X-100 in PBS, incubated with secondary antibodies for 1 hour at RT, followed by a subsequent wash with 0.1% Triton X-100 in PBS (4x), and mounted in ProLong Gold Antifade with DAPI (Molecular Probes). Images were acquired on a DeltaVision Elite deconvolution system (Applied Precision/GE Healthcare) using SoftWorx 6.0 software (Applied Precision/GE Healthcare) and a 100x/1.40 NA UPlanSApo objective (Olympus). Images are maximum intensity projections of deconvolved stacks and quantified using CellProfiler [16], and the average kinetochore intensity of BUB1 was calculated over the kinetochore intensity of CENP-C.

**Antibodies**

Primary antibodies used for immunofluorescence were rabbit-anti-BUB1 (Abcam; #ab9000) and guinea pig anti-centromere protein C (CENP-C; MBL; #PD030) and secondary antibodies goat anti–rabbit Alexa Fluor 488 (Molecular Probes; #A11034) and goat anti-guinea pig Alexa Fluor 647 (Molecular Probes; #A21450).

**SUPPLEMENTARY TABLES AND FIGURES**

***Table S1.*** *LOH results for BUB1 and BUB3.*

| **Gene** | **Germline mutation^a^** | **Family ID** | **Patient** | **LOH markers** |
| --- | --- | --- | --- | --- |
| *BUB1* | c.1965-1G>A | A | III.1 | n.a. |
| *BUB1* | c.2296G>A (p.E766K) | B | II.6, III.2 | D2S1888 (n.i., no LOH), D2S1889 (n.i., no LOH), D2S1892 (no LOH, n.i.) |
| *BUB1* | c.2473C>T (p.P825S) | C | III.4 | D2S1888 (n.i.), D2S1889 (no LOH), D2S1892 (no LOH) |
| *BUB3* | c.77C>T (p.T26I) | D | II.5 | D10S1483 (no LOH), D10S587 (no LOH), D10S1723 (n.i.) |

1. RefSeq Ensembl GRCh37: BUB1, NM_004336; BUB3, NM_004725

Abbreviations: LOH, loss of heterozygosity; n.a., not available information; n.i., non-informative

**Table S2.** Cytogenetic changes identified in lymphocytes from mutation carriers and controls.

| **Gene** | **Studied sample** | **Chromosomal alterations (N)** | | | |
| --- | --- | --- | --- | --- | --- |
|  |  | Normal | Loss | Gain | Structural |
| *BUB1* | Control 1 | 25 | 15 | 0 | 0 |
|  | Fam A (III.1) | 30 | 10 | 0 | 1 |
|  | Control 2 | 14 | 11 | 0 | 0 |
|  | Fam B (III.2) | 25 | 5 | 0 | 0 |
|  | Control 3 | 7 | 13 | 6 | 0 |
| *BUB3* | Fam D (II.5) | 16 | 0 | 0 | 0 |

Abbreviations: Fam, family; N, number of metaphases

**Table S3.** Genetic and phenotypic traits of the members of cancer families carrying unreported or rare (MAF<1%) BUB1 or BUB3 germline variants reported to date [17, 18, 19].

| **^a^Mutation** | **Cancer (age at diagnosis)** | **Hereditary CRC criteria** | **Functional evidence of deleteriousness** | **Publication** |
| --- | --- | --- | --- | --- |
| ***BUB1*** |  |  |  |  |
| 2q13 microdeletion | CRC (37) | Bethesda | Yes | de Voer [17] |
| ^b^c.46C>T; p.Q16* | Jejunum ca. (34); CRC x2 (40);  Renal cell ca. (44); Lung ca. x2 (45, smoker) | Amsterdam II | Disruptive mutation | de Voer [17] |
| c.46C>T; p.Q16* | Cancer-free (age?) |  |  |  |
| c.1375T>A; p.Ser459Thr | CRC (age?) | Amsterdam | n.a. | Broderick [18] |
| c.1965-1G>A; p.S655Rfs*32 | CRC (40); 25 colonic adenomas (40) | Attenuated polyposis | Yes / Disruptive mutation | Present study |
| c.2296G>A; p.E766K | CRC (65) | Amsterdam I | No | Present study |
| c.2296G>A; p.E766K | CRC (42) |  |  |  |
| c.2296G>A; p.E766K | CRC x2 (73); CRC (74) |  |  |  |
| c.2473C>T; p.P825S | CRC (44) | Bethesda | n.a. | Present study |
| c.2844delC; p.Q949Rfs*3 | CRC (31) | Amsterdam II | Disruptive mutation | de Voer [17] |
| ***BUB3*** |  |  |  |  |
| c.63G>C; p.K21N | CRC (32) | Bethesda | Yes | de Voer [17] |
| c.77C>T; p.T26I | CRC x2 (73); Prostate ca.(70); 22 colonic adenomas | Attenuated polyposis / Amsterdam I (no cosegregation) | n.a. | Present study |
| c.446G>A; p.R149Q | CRC (38); 4 colon adenomas (59-61) | Bethesda | n.a. | de Voer [17] |
| c.576+1G>A; p.? | Pancreatic ductal ca. (69) | No | Disruptive mutation | Shindo [19] |
| c.790T>C; p.F264L | CRC(29); Lung ca. (44, smoker) | Bethesda | Yes | de Voer [17] |

1. RefSeq GRCh37: *BUB1* NM_004336, *BUB3* NM_004725
2. This patient also carried an *MLH1* pathogenic mutation (c.453+1G>T) associated with Lynch Syndrome.

Abbreviations: ca., cancer; CRC, colorectal cancer; n.a., not available.

**Table S4.** Primers used in the study.

| **Analysis** | **Forward (5’-3’)** | **Reverse (5’-3’)** | **Product (bp)** |
| --- | --- | --- | --- |
| Mutation screening | |  |  |
| BUB1 Ex1 | GAGGAGCTACTGGCTCAAGG | ACACATTCCAAACCCAGGAA | 356 |
| BUB1 Ex2-3 | TCTTTTGCTTGTAGAAATTATCCCTAA | GCCCAGCCCCTACATACTTT | 533 |
| BUB1 Ex4 | AGCTGGCTAACTCCTCACCA | GGATTTCCCTGTACAAATTGC | 369 |
| BUB1 Ex5 | TCGAAGCAACTCTAAAATGCAA | CTCCCAAAGTGCTGGGATTA | 328 |
| BUB1 Ex6 | ACTGGAAGGATCAGGGGAAT | GGTGAGCTGTGTGGGAGAAT | 377 |
| BUB1 Ex7-8 | CTTCCCCTCAAGCATTTTCA | TCTGGGCTTCTGATAGGATGA | 567 |
| BUB1 Ex9 | AAGCTGTGGGCAGGTAAAAA | ATCAAACTGCCATTCCTGCT | 387 |
| BUB1 Ex10 | CAGATGCCATGCTTTGAGAA | GGAGCAATGTTTTGCCACTT | 517 |
| BUB1 Ex11 | CTCCCAAAGTGCTGGGATTA | CCACAGCCACAAAATACACCT | 459 |
| BUB1 Ex12-13 | TCCAGACCAACCACTCAATCT | ATGCATTCCCTGCCTTTATG | 933 |
| BUB1 Ex14-15 | GATATGTGGCAGGGACTCGT | TAAAGCGGATGGGTTGTAGG | 887 |
| BUB1 Ex16 | CCAGGGGTTGACATCTGTTT | TGGGTTGGACCTTATTTCCTT | 593 |
| BUB1 Ex17 | GCCTTGCCTCAAACTACTGG | TGAAGAGAATGGCAGAACCA | 414 |
| BUB1 Ex18 | AACCCACTCCTGACATTTCCT | CCTGGCACGTAGTAGGCTGT | 777 |
| BUB1 Ex19 | TTCCAGTTGAGTGGAGCAAA | TAGTCCCAACAGCAAGCACA | 617 |
| BUB1 Ex20 | CCAATTTGAGGGCCATTCTA | TGAACTGTTGCTGCAATGTG | 698 |
| BUB1 Ex21-23 | CCACATTGCAGCAACAGTTC | TCTGATAAGCGCAACACAGAA | 958 |
| BUB1 Ex24 | GACATGTTAGTGGGGCGATT | CAGTGGGGAGAGAGAAGTGG | 460 |
| BUB1 Ex25 | AGCCTGTGGAAGTCAGATGC | AAATGCTTGCATCCCAGAAG | 507 |
| BUB3 Ex2 | GCAAGCGCAGAGTCTCCT | GAAAAGAAACGCTCCTCACG | 395 |
| BUB3 Ex3 | GCAGGTATTGAACTATACCT | CACTAGCCAAGAAAACAAACTGG | 309 |
| BUB3 Ex4 | TGAGCACCTAGCCTGTACCA | AATCACCCCAAAACCATCAA | 586 |
| BUB3 Ex5 | GAGAGCATCATGGGTTTGCT | GCAGGTATTGAACTATACCT | 372 |
| BUB3 Ex6-7 | TGTGGGTTGAATTTGGGAAT | TATCGAGCAGGCATCAACAA | 775 |
| BUB3 Ex8 | GGCCATTTTGATCTCATGCT | CACTTGCCCTCCAATTTTGT | 307 |
| Loss of heterozigosity | |  |  |
| BUB1 D2S1888 | FAM-TTTGAAGTTTGGTGTCTGTGTAA | TGAAGTCCCTTGGAAATGTT | 85 |
| BUB1 D2S1889 | FAM-GTTCCAAATCCTTGCC | AGCTTGCTTAAATTACCATTTT | 207 |
| BUB1 D2S1892 | FAM-TCCTAACTCTGAAATGCTAAAGACA | AGCTCTGGCAGGGAGA | 229 |
| BUB1 Ex19_FFPE | TGGGCTTTCTAAACCAGTGAG | TTCAGTCTTGGGCTTGATGG | 82 |
| BUB1 EX21_FFPE | GGGATAGAGGTTGCTTTATTGC | TGTAGAATTCCCAGGGGTTG | 110 |
| BUB3 D10S1483 | FAM-CAATGCTATCCCGGCTATG | TCAAGACTGCAAGCGTGT | 144 |
| BUB3 D10S587 | FAM-CCCAGATTCATGGCTTTC | TTCTGCTGACACGGGC | 176 |
| BUB3 D10S1723 | FAM-GCCTTCATTTGCATAGGG | CATGCTGAGACCCAGTG | 164 |
| BUB3 Ex2_FFPE | GACCGGTTCTAACGAGTTCAA | AGGAGACAAGCAGGAACTGG | 98 |
| Methylation | |  |  |
| BUB1 promoter | AGTGGGAGGAGTTATTGGTTTAAG | AACACTTACTAAAAAACATTTTCC | 247 |
| Splicing analyses | |  |  |
| BUB1 c.1965-1G>A | AGAGCCCAGGAGACTTCACA | ATCTCCCTGGGTAGCTTCGT | 641 |
| BUB1 c.2296G>A | ACCCATGGGATGATAAGCTG | ATCTCCCTGGGTAGCTTCGT | 206 |
| BUB3 c.77C>T | GACCGGTTCTAACGAGTTCAA | TTCCAGTGACCATCACATTCA | 341 |

**
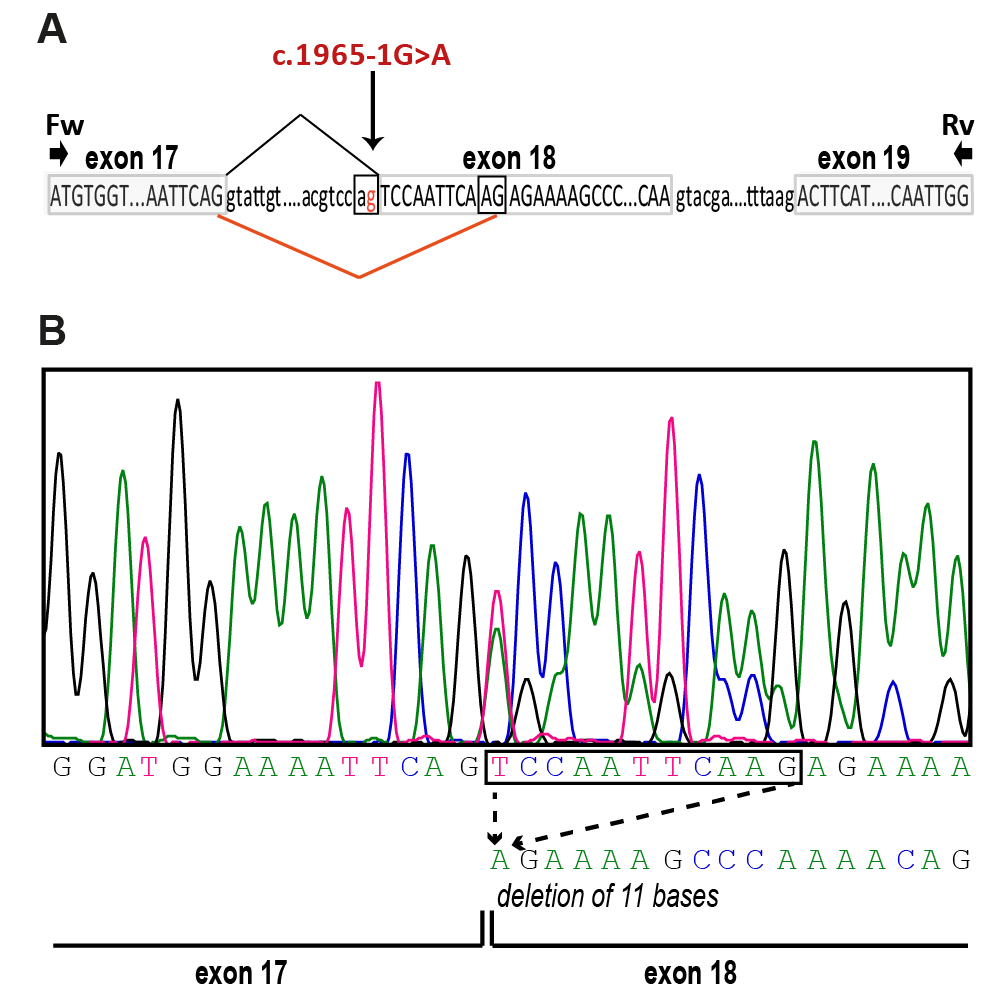
**

**Figure S1.** Characterization of *BUB1* c.1965-1G>A. A) Schematic representation of the affected region and location of the mutation, the affected acceptor splicing site and the new acceptor site (AG) located within exon 18. The location of the primers used for cDNA amplification is also indicated. B). Sequencing of the cDNA PCR amplification product revealed the deletion of the first 11 bases of exon 18 in one allele.

**REFERENCES**

1. Bellido F, Pineda M, Aiza G, Valdés-Mas R, Navarro M, Puente DA, et al. POLE and POLD1 mutations in 529 kindred with familial colorectal cancer and/or polyposis: review of reported cases and recommendations for genetic testing and surveillance. Genet Med. 2016, 18:325-32.

2. Puente XS, Pinyol M, Quesada V, Conde L, Ordóñez GR, Villamor N, et al. Whole-genome sequencing identifies recurrent mutations in chronic lymphocytic leukaemia. Nature. 2011, 475:101-5.

3. Kumar P, Henikoff S, Ng PC. Predicting the effects of coding non-synonymous variants on protein function using the SIFT algorithm. Nat Protoc. 2009, 4:1073-81.

4. Adzhubei IA, Schmidt S, Peshkin L, Ramensky VE, Gerasimova A, Bork P, et al. A method and server for predicting damaging missense mutations. Nat Methods. 2010, 7:248-9.

5. González-Pérez A, López-Bigas NCP. Improving the assessment of the outcome of nonsynonymous SNVs with a consensus deleteriousness score, Condel. Am J Hum Genet. 2011, 88:440-9.

6. Tavtigian SV, Deffenbaugh AM, Yin L, Judkins T, Scholl T, Samollow PB, et al. Comprehensive statistical study of 452 BRCA1 missense substitutions with classification of eight recurrent substitutions as neutral. J Med Genet. 2006, 43:295-305.

7. Mathe E, Olivier M, Kato S, Ishioka C, Hainaut P, Tavtigian SVCP. Computational approaches for predicting the biological effect of p53 missense mutations: a comparison of three sequence analysis based methods. Nucleic Acids Res. 2006, 34:1317-25.

8. Dehouck Y, Kwasigroch JM, Gilis D, Rooman MCP. PoPMuSiC 2.1: a web server for the estimation of protein stability changes upon mutation and sequence optimality. BMC Bioinformatics. 2011, 12:151.

9. Yin S, Ding F, Dokholyan NV. Eris: an automated estimator of protein stability. Nat Methods. 2007, 4:466-7.

10. Capriotti E, Fariselli P, Casadio RCP. I-Mutant2.0: predicting stability changes upon mutation from the protein sequence or structure. Nucleic Acids Res. 2005, 33:W306-10.

11. Parthiban V, Gromiha MM, Schomburg DCP. CUPSAT: prediction of protein stability upon point mutations. Nucleic Acids Res. 2006, 34:W239-42.

12. Vázquez M, Valencia A, Pons TCP. Structure-PPi: a module for the annotation of cancer-related single-nucleotide variants at protein-protein interfaces. Bioinformatics. 2015, 31:2397-9.

13. Borràs E, Pineda M, Brieger A, Hinrichsen I, Gómez C, Navarro M, et al. Comprehensive functional assessment of MLH1 variants of unknown significance. Hum Mutat. 2012, 33:1576-88.

14. Yost S, de Wolf B, Hanks S, Zachariou A, Marcozzi C, Clarke M, et al. Biallelic TRIP13 mutations predispose to Wilms tumor and chromosome missegregation. Nat Genet. 2017, 49:1148-51.

15. Drost J, van Jaarsveld RH, Ponsioen B, Zimberlin C, van Boxtel R, Buijs A, et al. Sequential cancer mutations in cultured human intestinal stem cells. Nature. 2015, 521:43-7.

16. Carpenter AE, Jones TR, Lamprecht MR, Clarke C, Kang IH, Friman O, et al. CellProfiler: image analysis software for identifying and quantifying cell phenotypes. Genome Biol. 2006, 7:R100.

17. de Voer RM, Geurts van Kessel A, Weren RD, Ligtenberg MJ, Smeets D, Fu L, et al. Germline mutations in the spindle assembly checkpoint genes BUB1 and BUB3 are risk factors for colorectal cancer. Gastroenterology. 2013, 145:544-7.

18. Broderick P, Dobbins SE, Chubb D, Kinnersley B, Dunlop MG, Tomlinson I, et al. Validation of Recently Proposed Colorectal Cancer Susceptibility Gene Variants in an Analysis of Families and Patients-a Systematic Review. Gastroenterology. 2017, 152:75-7 e4.

19. Shindo K, Yu J, Suenaga M, Fesharakizadeh S, Cho C, Macgregor-Das A, et al. Deleterious Germline Mutations in Patients With Apparently Sporadic Pancreatic Adenocarcinoma. J Clin Oncol. 2017:JCO2017723502.
